# Commentary on “Synaptic function is modulated by LRRK2 and glutamate release is increased in cortical neurons of G2019S LRRK2 knock-in mice”

**DOI:** 10.3389/fncel.2014.00351

**Published:** 2014-10-28

**Authors:** Elena Popugaeva, Ilya Bezprozvanny

**Affiliations:** ^1^Laboratory of Molecular Meurodegeneration, Department of Medical Physics, St. Petersburg State Polytechnical UniversitySt. Petersburg, Russia; ^2^Department of Physiology, UT Southwestern Medical Center at DallasDallas, TX, USA

**Keywords:** Parkinson's disease, LRRK2, G2019S, synapse function, glutamate transmission

Parkinson's disease (PD) is the most common form of age related motor disorder (Hsu et al., 2010; Ferree et al., 2012). Mutations in leucine-rich repeat kinase 2, LRRK2, gene are considered to be genetic determinants of PD. Among them G2019S is the most prevalent amino acid substitution mutation in LRRK2 and accounts for 1–2% of sporadic PD cases (Healy et al., 2008). LRRK2 is involved in many signaling pathways and its role is different in different cell types. In case of PD the main interest lies in understanding the function of LRRK2 in neuronal physiology.

The study of Beccano-Kelly et al. attempts to investigate the role of LRRK2 in synaptic physiology in the context of loss of function and gain of function. Authors compared and contrasted obtained results on normal LRRK2 functions to effects of G2019S mutant LRRK2. To perform experiments they used primary cortical cultures prepared from LRRK2 transgenic overexpressing (OE), knock-out (KO), and knock-in G2019S (KI) mice. Beccano-Kelly et al. report that LRRK2 modulates synaptic function via regulation of glutamatergic activity. This result is a continuation of earlier findings obtained by other research groups in *Drosophila* model (Lee et al., 2010; Matta et al., 2012) and in mammalian cortical cultures (Piccoli et al., 2011; Parisiadou et al., 2014). Mentioned studies are contradictory in relation to whether deletion of LRRK2 upregulates, or downregulates, glutamatergic synaptic transmission. Beccano-Kelly et al. found out that LRRK2 knock out leads to reduced glutamatergic activity. The current study and previous reports from other groups do not uncover the mechanism of LRRK2 mediated synaptic function. This point should be investigated in the future studies. The most interesting result of Beccano-Kelly et al. was that in the absence of any change to synapse density glutamate release was markedly elevated in knock-in cultures. This indicated that physiological levels of G2019S LRRK2 elevate probability of release. Next observation was that the phosphorylation of synapsin 1 was significantly reduced in KI neurons. Based on obtained results authors concluded that perturbations to the presynaptic release machinery and elevated synaptic transmission are early neuronal effects of LRRK2 G2019S.

Taken together, the study of Beccano-Kelly et al. presents novel data about the role of normal and G2019S mutated LRRK2 in regulation of synaptic transmission. On the one hand, discovering the signaling mechanism underlying LRRK2-mediated regulation of synaptic function could lead to the development of PD-preventing therapies.

## Conflict of interest statement

The authors declare that the research was conducted in the absence of any commercial or financial relationships that could be construed as a potential conflict of interest.
